# The Effect of ASIC3 Knockout on Corticostriatal Circuit and Mouse Self-grooming Behavior

**DOI:** 10.3389/fncel.2019.00086

**Published:** 2019-03-12

**Authors:** Wei-Li Wu, Sin-Jhong Cheng, Shing-Hong Lin, Yu-Chia Chuang, Eagle Yi-Kung Huang, Chih-Cheng Chen

**Affiliations:** ^1^Institute of Biomedical Sciences, Academia Sinica, Taipei, Taiwan; ^2^Department of Physiology, College of Medicine, National Cheng Kung University, Tainan, Taiwan; ^3^Division of Biology and Biological Engineering, California Institute of Technology, Pasadena, CA, United States; ^4^Dana Farber Cancer Institute and Department of Neurobiology, Harvard Medical School, Boston, MA, United States; ^5^National Defense Medical Center, Department of Pharmacology, Taipei, Taiwan; ^6^Taiwan Mouse Clinic—National Comprehensive Mouse Phenotyping and Drug Testing Center, Academia Sinica, Taipei, Taiwan

**Keywords:** ASIC3, self-grooming, corticostriatal circuit, parvalbumin, proprioception

## Abstract

Stereotypic and/or repetitive behavior is one of the major symptoms of autism spectrum disorder (ASD). Increase of self-grooming behavior is a behavioral phenotype commonly observed in the mouse models for ASD. Previously, we have shown that knockout of acid-sensing ion channel 3 (ASIC3) led to the increased self-grooming behavior in resident-intruder test. Given the facts that ASIC3 is mainly expressed in the peripheral dorsal root ganglion (DRG) and conditional knockout of ASIC3 in the proprioceptors induced proprioception deficits. We speculate a hypothesis that stereotypic phenotype related to ASD, pararalled with striatal dysfunction, might be caused by proprioception defect in the peripheral sensory neuron origin. Herein, we investigate in depth whether and how ASIC3 is involved in the regulation of self-grooming behavior. First, we observed that *Asic3* null mutant mice exhibited increased self-grooming in social interaction during juvenile stage. Similarly, they displayed increased self-grooming behavior in a novel cage in the absence of cagemate. To further understand the mechanism by which ASIC3 affects grooming behavior, we analyzed neurochemical, neuropathological and electrophysiological features in the dorsal striatum of *Asic3* null mutant mice. Knockout of *Asic3* increased dopamine (DA) activity and phospho-ERK immunoreactivities in the dorsal striatum. Furthermore, we detected a lower paired-pulse ratio (PPR) and impaired long-term potentiation (LTP) in corticostriatal circuits in *Asic3* null mutant mice as compared with wild-type (WT) littermates. Moreover, knockout of *Asic3* altered the medial spiny neurons in the striatum with defects in presynaptic function and decrease of dendritic spines. Lastly, genetic ablation of *Asic3* specifically in parvalbumin-positive (PV^+^) cells resulted in the increase of self-grooming behavior in mice. These findings suggest knockout of *Asic3* in the PV^+^ neurons alters grooming behavior by co-opting corticostriatal circuits.

## Introduction

Self-grooming is an innate behavior that can be observed across multiple species (Kalueff et al., 2016). Excessive self-grooming behavior in rodents is a significant stereotypic and/or repetitive behavior that could be directly linked to psychiatric disorders such as autism spectrum disorder (ASD) and obsessive-compulsive disorder (OCD; Silverman et al., 2010; Langen et al., 2011; Ting and Feng, 2011; Shepherd, 2013). In addition, stereotypic/repetitive behavior is one of the major symptoms of ASD (Zandt et al., 2007; Jacob et al., 2009), with their multigenic inheritance and similar affected neural circuits (Abrahams and Geschwind, 2008; Bill and Geschwind, 2009; Jacob et al., 2009; Lewis and Kim, 2009). Thus, grooming behavior in rodents is an applicable indication with which researchers can unveil candidate genes dominating stereotypic behaviors.

Grooming behavior in rodents directly involves the animal’s own face, whiskers, and body. In 1986, Berridge and Fentress described rat postprandial grooming behavior and further named the ritually and stereotypic chain sequences of grooming behavior “action syntax” (Berridge and Fentress, 1986; Kalueff et al., 2007). The neural circuits underlying self-grooming behavior had been extensively investigated. Most of the evidence indicate that corticostriatal circuits is involved in the control of self-grooming behavior (Burguière et al., 2013; Kalueff et al., 2016).

Acid-sensing ion channel 3 (ASIC3) is a voltage-independent cation channel sensitive to mild extracellular acidification and is abundantly expressed in the peripheral nervous system (PNS; Wu et al., 2012). Due to the acid-sensitivity and predominant expression in pain-sensing neurons (nociceptors), ASIC3 is evidenced playing an important role in pain associated with tissue acidosis (Sun and Chen, 2016). Interestingly, ASIC3 is also expressed in proprioceptors, which are low-threshold mechanoreceptors mediate neurosensory mechanotransduction of muscle afferents and proprioceptive behaviors (Lin et al., 2016). Accumulating evidences have shown that sensory symptoms are found as early as 6 months in infants later diagnosed with autism and are predictive of social-communication deficits and repetitive behaviors in childhood (Robertson and Baron-Cohen, 2017). Recent studies also show peripheral mechanosensory neuron dysfunction could contribute to the development of ASD in mouse models (Orefice et al., 2016). However, although the causal effect of aberrant tactile sensitivity and ASD has been established, the specific mechanosensory neuron subtypes and molecular determines that contribute to the development of ASD are still not clear.

Our previous studies found that *Asic3* knockout (*Asic3*^−/−^) mice showed heightened self-grooming during resident-intruder test (Wu et al., 2010). Moreover, *Asic3*^−/−^ mice showed lower serotonin turnover rate in the striatum, midbrain and brainstem after social interaction, which implicates dysregulation of monoamine metabolism in these brain regions (Wu et al., 2009). Since there is no evidence that ASIC3 is functionally expressed in the central nervous system (CNS), we hypothesized that the dysfunction of proprioception could cause the self-grooming phenotypes in *Asic3*^−/−^ mice. This hypothesis aligns well with the observation of somatosensory and tactile dysfunction in ASD (Marco et al., 2012; Puts et al., 2014).

Here, we investigated the underlying neural mechanism of how ASIC3 controls grooming behavior in mice. By combining genetic manipulation, behavior analysis, neuropathology, neurochemistry, and electrophysiology, we demonstrated that mutation of proprioceptive ASIC3 may result in increased grooming behavior in mice with dysregulation of the corticostriatal circuit.

## Materials and Methods

### Mice

Constitutive *Asic3*^−/−^ mice were generated and bred in a CD1 strain as described (Chen et al., 2002; Wu et al., 2009, 2010). Unless specifically mentioned, *Asic3*^−/−^ and *Asic3*^+/+^ mice were littermates derived from *Asic3^+/−^* mice. Mice carrying the floxed alleles of *Accn3* (*Asic3*^f/f^) were generated as described (Lin et al., 2016). Basically, the exon1 of *Accn3*, which contains the translation initiation codon and one-quarter of the coding sequence, was flanked by two loxP sites. *Pv-Cre* (B6;129P2-*Pvalbtm1(cre)Arbr*/J) mice were from the Jackson Laboratory (Bar Harbor, ME, USA). *Na_v_1.8-Cre* mice were generated as described (Stirling et al., 2005). Cre mice were crossed with *Asic3*^f/f^ mice to generate conditional *Asic3*-knockout mice (*Cre*^+^/*Asic3*^f/f^). Conditional knockout *Pv-* (or *Na_v_1.8*)-*Cre*^+^/*Asic3*^f/f^ mice and control *Cre*^−^/*Asic3*^f/f^ mice were offspring from *Pv* (or *Na_v_1.8*)-*Cre*^+^/*Asic3*^f/f^ mice intercrossed. The expression of *Pv-Cre* and *Na_v_1.8-Cre* was examined by crossing mice with *ROSA26R* mice in our prior work (Lin et al., 2016). Mice were group-housed with a 12-h/12-h light/dark cycle (lights on 8:00–20:00) at 23°C and 40%–70% humidity. All behavior experiments were performed at 13:00–18:00 daily. This study was carried out in accordance with the recommendations of IACUC of Academia Sinica. The protocol was approved by the IACUC of Academia Sinica.

### Genotyping of *Asic3* Conditional Knockout Mice and Verification of Excised Allele

Genomic DNA from tails of mice was genotyped by PCR for the presence of the *Asic3* floxed allele, excised floxed allele, generic *Cre* allele, and *Na_v_1.8-Cre* allele. For the *Cre* allele in *Na_v_1.8-Cre* mice, the primer sequences were forward, 5′-TGTAGATGGACTGCAGAGGATGGA-3′, and reverse, 5′-AAATGTTGCTGGATAGTTTTTACTGCC-3′ (420 bp). For the generic Cre in PV-Cre experiments, the primer sequences were forward, 5′-GCGGTCTGGCAGTAAAAACTATC-3′, and reverse, 5′-GTGAAACAGCATTGCTGTCACTT-3′ (102 bp). To screen for the presence of the downstream loxP fragment in intron 1 of the mouse *Accn3* gene, the ASIC3 floxed allele, the primer sequences were forward, 5′-GATTTGTCACTGCCATGGTG-3′, and reverse, 5′-GGCAGATACTCCTCCTGCTG-3′. Upon Cre-mediated floxed allele excision, the forward primer sequence 5′-CTCGAGGCCCACATAACTTCG-3′ corresponding to the upstream loxP fragment in the 5′ untranslated region was combined with the same reverse primer used for floxed allele genotyping to confirm the excision of the floxed *Accn3* exon 1. The fragment size for the wild-type (WT), ASIC3 conditional knockout, and ASIC3 excised alleles was 400, 450, and 306 bp, respectively.

### Measurement of Striatal Dopamine Activity

The procedure was as for our previous work (Wu et al., 2009). Briefly, subregions of brain were dissected according to the mouse brain atlas. Tissue was immediately frozen on dry ice and stored in −80°C until use. On the day of analysis, tissue was homogenized in 0.1 mM oxalic acid and then centrifuged at 13,000× *g* for 40 min at 4°C. Supernatants were removed and filtered through a 0.22-μm syringe filter (Millipore, Bedford, MA, USA) for high-performance liquid chromatography (HPLC). The HPLC system consisted of a reverse-phase C18 column (MD-150, RP-C-18, 5 μM, length: 15 cm. ESA, Chelmsford, MA, USA), a high-pressure pump (PM-80, Bioanalytical Systems, West Lafayette, IN, USA) connected with an electrochemical detector (ECD; LC-4C) and coupled to a reference electrode (Ag/AgCl) and a glassy carbon working electrode, which was set at +750 mV (Bioanalytical Systems, West Lafayette, IN, USA). Under an isocratic condition, the solvent of the mobile phase, consisting of 75 mM NaH_2_PO_4_.H_2_O, 1.7 mM 1-Octanesulfonic acid, 25 μM EDTA, 0.72 mM TEA and 10% acetonitrile (pH 3.0; solution degassed for 10 min before use), was pumped and circulated at a flow rate of 1 ml/min in the system. An amount of 20 μl of each sample was injected into the HPLC-ECD for analysis. The final concentrations of dopamine (DA) and its metabolite 3,4-dihydroxyphenylacetic acid (DOPAC), and homovanillic acid (HVA) in tissue samples were determined by use of CSW32 software (DataApex, Soubêžnâ, Czech Republic). The standard curve was created with 500, 100, 50, 10, and 5 nM of each chemical. The standard curve was used only with *R*^2^ > 0.99 under linear regression. To calibrate the sample peak, each sample was calibrated with external standards, which were freshly prepared and injected every five sample runs. The index of DA turnover rate was calculated as (DOPAC+HVA)/DA.

### Immunohistochemistry

Mice under anesthesia were transcardially perfused with 30 ml phosphate buffered saline (PBS) and then 30 ml 4% paraformaldehyde. Brains were sampled and post-fixed in 4% paraformaldehyde overnight, then dehydrated in 30% sucrose for 3 days with the buffer changed each day. Brains were embedded in OCT and rapidly frozen on dry ice, then stored at −80°C until sectioning in 50-μm sections by use of a cryostat (CM3050S; Lecia Microsystems, Wetzlar, Germany) and collected in tris-buffered saline (TBS). For phosphorylated extracellular signal-regulated kinase 1/2 (pERK1/2) staining, free-floating sections were first washed with TBS three times and immersed in 0.6% H_2_O_2_ at room temperature for 30 min to block endogenous peroxidase, immersed in blocking solution (1% bovine serum albumin and 0.1% Triton X-100, 0.02% sodium azide in PBS) at room temperature for 1 h, then incubated with rabbit anti-pERK1/2 antibody (1:500 in blocking solution; #9101; Cell Signaling, Danvers, MA, USA) at 4°C overnight. The next day, sections were incubated with biotinylated anti-rabbit secondary antibody (1:500; Vectastain ABC Kit, Vector Labs, Burlingame, CA, USA) for 1 h at room temperature, then with ABC reagent (1:500; Vectastain ABC kit, Vector Labs, Burlingame, CA, USA) for 1 h at room temperature. Finally, sections were reacted with DAB/nickel solution (1 mg/ml DAB, 4 mg/ml D(+)-glucose, 1.6 mg/ml NH_4_Cl, 5 ml nickel solution [0.03 g/ml nickel in 0.1 M sodium acetate pH 6]) and glucose oxidase in TBS. Sections were washed with TBST three times in each step. Free-floating sections were then attached in order on gelatin-coated coverslips according to the mouse brain atlas. Photos were taken at 10× magnification. Quantification of pERK1/2-positive cells involved Adobe Photoshop and was further analyzed by ImageJ Software.

### Golgi Staining

Golgi staining involved use of the FD Rapid GolgiStain Kit (PK-401, FD NeuroTechnologies, Ellicott City, MD USA). Briefly, age-matched mice under anesthesia were transcardially perfused with saline. Mouse brains were excised, rinsed in double distilled water, and immersed in impregnation solution A+B (1:1) at room temperature. The impregnation solution was replaced the next day and then stored at room temperature for 14 days. Then brains were transferred to solution C and stored at 4°C in the dark for another 6 days. The solution was replaced once on the next day. After 6 days, brains were transferred to 30% sucrose solution at 4 for 1 day, sliced into 150-μm thick sections by use of a vibratome (St. Louis, MO, USA) and collected in milli-Q water. Sections were stained with solution D+E for 10 min and washed with milli-Q water 2–3 times × 2 min between each step. Sections were mounted on gelatin-coated coverslips and dehydrated in ethanol gradient (50%–100%). Slides were immersed in xylene for 1 h and covered with Entellan (Merck, Darmstadt, Germany).

The stained MSNs with oil immersion were photographed at 1,000× magnification. For each mouse, we randomly photographed 9–24 MSNs in the striatum CPu (Bregma AP: 0.38 ~ −0.1 mm), where the pERK expression was higher in *Asic3*^−/−^ than *Asic3*^+/+^ mice (*n* = 6 for *Asic3*^+/+^ mice; *n* = 5 for *Asic3*^−/−^ mice). About 20~50 photos were taken of each neuron in different focus ranges to reveal the integrity of each MSN and its dendritic spine. The images were then merged together by use of ImageJ (NIH, Bethesda, MD, USA). The person who counted the number of dendritic spines was blinded to mouse genotype. We sampled the dendrites at 60–80 μm from the cell body, which is considered to contain stable dendritic spine density (Cheng et al., 1997).

### Extracellular Field Recording

Acute brain slices were prepared from 8- to 10-week-old WT and *Asic3* mutant mice for extracellular field recording. The mice were anesthetized with isoflurane (1.5–2% in 100% O_2_) and decapitated, and brains were quickly removed and placed in ice-cold artificial cerebrospinal fluid (ACSF) containing (in mM): 119 NaCl, 2.5 KCl, 1.3 MgSO_4_, 26.2 NaHCO_3_, 1 NaH_2_PO_4_, 2.5 CaCl_2_, and 11 glucose; the pH was maintained at 7.4 by carbogenating and oxygenating the solution with 95% O_2_ and 5% CO_2_ and the osmolarity of ACSF was adjusted to 300 mOsm. The brains were rapidly blocked for sagittal sectioning at 450 μm thickness by use of a vibroslicer (DTK-1000, D.S.K., Osaka, Japan). Slices containing the dorsal striatum were kept in oxygenated ACSF (95% O_2_ and 5% CO_2_) at room temperature (24–25°C) to allow recovery for at least 90 min before the recording commenced.

Slices after 90 min recovery were transferred to an immersion-type recording chamber and constantly perfused with oxygenated ACSF at 1.5–2.0 ml min^−1^. The recording chamber was mounted to an upright microscope (BX50WI, Olympus Optical, Tokyo), equipped with an infrared-differential interference contrast microscopy video. The corpus callosum was clearly identified under low magnification. A borosilicate glass recording electrode filled with 3 M NaCl was placed in the dorso-lateral striatum and a bipolar stainless steel electrode (FHC, St. Bowdoin, ME, USA) was placed on the inner border of the corpus callosum between the cortex and dorsolateral striatum to predominantly activate corticostriatal fEPSPs. To evoke fEPSPs, the slices were constantly perfused with ACSF containing 0.1 mM picrotoxin, a GABA (c-aminobutyric acid) A receptor antagonist, to block contaminating responses from intra-striatal GABAergic circuitry. Corticostriatum fEPSPs were evoked with 40-μs step-increasing stimulation at 0.2 mA intensity and 0.1 Hz to measure the input–output relationship from cortico-striatal synapses. For paired-pulse stimulation, twin pulses of the same intensity were given at 0.067 Hz, with the inter-pulse interval set to 50 and 100 ms. Paired-pulse ratio (PPR) was measured as the ratio of the second EPSP to the first EPSP for responses to paired pulse stimulation. For long-term potentiation (LTP) experiments, baseline responses were monitored to assure stable EPSP for a minimum of 10 min, then three trains (separated by 60 s) of 100 stimuli at 100 Hz were applied for LTP induction. The slopes of fEPSPs were measured and normalized to the averaged value of the baseline responses; the averaged fEPSP slopes recorded 21 ~ 30 min after the LTP induction were used for statistical comparison. All recordings were performed at room temperature.

### Striatal Slice Preparation for Whole-Cell Recording

Whole-cell mEPSC recordings in the striatal MSNs were conducted in 300-μm-thick acute brain slices from 8- to 10-week-old WT and *Asic3* mutant mice. The slice was transferred into a holding chamber mounted to an upright microscope (BX50WI, Olympus Optical, Tokyo), equipped with an infrared-differential interference contrast microscopic video. The MSN whole-cell patch-clamp recordings were obtained under visual guidance with patch pipettes pulled from borosilicate glass (1.5-mm outer diameter, 0.32-mm wall thickness; G150F-4, Warner Instruments, Hamden, CT, USA). The patch electrodes had a resistance of 3–5 MΩ when filled with a solution consisting of (in mM): 131 K-gluconate, 20 KCl, 10 HEPES, 0.2 EGTA, 8 NaCl, 2 ATP, 0.3 GTP, and 6.7 biocytin; the pH was adjusted to 7.2 by KOH and osmolarity to 300~305 mOsm. Whole-cell patch-clamp recordings involved use of an Axopatch MultiClamp 700B system (Axon instruments) at room temperature. To record mEPSCs, neurons were held at −70 mV in the voltage clamp mode in the presence of 1 mM TTX (from Tocris), and the series resistance (Rs) was constantly monitored by applying a voltage pulse of 2 mV; the Rs was usually <20 M and was not compensated. Data were discarded when Rs varied by more than 20% of its original value during the recording. All signals were low-pass-filtered at the corner frequency of 1 kHz and then digitized at 10 kHz with use of a Micro 1401 interface (Cambridge Electronic Design, Cambridge, UK). Data were collected by use of Signal 3.0 and Spike2 software (Cambridge Electronic Design).

### RNA Extraction and Gene Expression Analysis

RNA extraction and quantitative real-time polymerase chain reaction (qRT-PCR). RNA extraction of brain subregion samples was conducted with RNeasy Mini Kit (Qiagen) with on column DNase digestion (Qiagen), based on the manufacturer’s protocol. The RNA concentration and quality were measured by NanoDrop 1000 (Thermo Scientific, Waltham, MA, USA). Total 0.6 μg RNA from each sample was reverse transcribed in 20 μl total volume using the iScript cDNA synthesis kit (Bio-Rad).

Gene expression of brain subregions was measured using FastStart Universal SYBR Green Master with Rox (Roche) and analyzed using ABI 7500 RT PCR System (Life Technologies, Carlsbad, CA, USA). Gene expression was normalized to gapdh mRNA. Data are presented as fold-change in gene expression in each group relative to control group. The primers of *Asic1a*, *Asic1b*, *Asic2a*, *Asic2b*, and *Asic4* were adapted from previous study (Schuhmacher and Smith, 2016). *Asic3* primers were designed based on Primer-BLAST (NCBI, NIH). The primer sequence for *Asic3* were forward, 5′-TATGTGGCTCGGAAGTGCGGAT-3′, and reverse, 5′-CAGACACAAGTGTCCTTTCGCAG-3′.

### Grooming Behavior During Reciprocal Social Interaction

The procedure was the same as in our previous work (Wu et al., 2009). Briefly, male mice 5 weeks old were isolated in a cage for 3.5–5 h. After isolation, the testing mouse was transferred to a new cage. An age-matched male stranger mouse was introduced into the cage. The interaction between two mice was recorded by use of a video camera for 15 min. The number of stereotypic behaviors (rearing, digging, and self-grooming) was manually counted by use of ETHOM software as described (Shih and Mok, 2000; Wu et al., 2009, 2010).

### Cross-Fostering

The procedure of cross-fostering offspring was previously described (Wu et al., 2009). Briefly, we set up timed mating for *Asic3^+/+^* and *Asic3*^−/−^ female mice to achieve pregnancy on the same date. Pregnant female mice were single housed and provided nesting material until the offspring were born. At P0, the dams were temporally moved to another cage. We gently transferred the whole litter to the opposite genotype’s dam without moving the nest. Then we put the dams back to their original cages and let them rear the cross-fostered offspring. The litter size was culled to 10–12 pups per litter. The offspring were weaned until P21.

### Grooming Behavior Test

The protocol was previously described (McFarlane et al., 2008). Mice were gently placed in a new empty cage without bedding. The total recording time was 20 min. The first 10 min was considered a habituation period, and the second 10 min was considered a testing period. The spontaneous grooming behavior was filmed by use of a CCD-computer imaging system (IR-H18D5PA, AGE Technology, Taoyuan, Taiwan) and saved in MPG format by a hardware encoder (MPB680III, Upmost Technology, Taipei). The film was then analyzed by use of HomeCageScan (Cleversys Inc, Reston, VA, USA).

### Digging Behavior

The protocol was as described by Deacon (2006). We first filled the clean cage with 5 cm wood bedding and gently flattened the bedding. Mice were placed in the cage for 3 min. The bedding was reused unless it was dirty. The digging behavior was filmed by use of a CCD-computer imaging system as described above. The total duration of digging behavior was manually analyzed by use of ETHOM (Shih and Mok, 2000).

### Statistical Analysis

All data are presented as mean ± SEM. The grooming behaviors in the social interaction test or cross-fostering test and digging behavior as well as striatum DA turnover rate, pERK1/2 staining and dendritic spine density were compared by two-tailed unpaired Student’s *t*-test. The grooming test was compared by two-way analysis of variance (ANOVA) followed *post hoc* Bonferroni analysis. Two-tailed paired *t-test* was used to compare responses before and after LTP induction. Analysis of mEPSC activity, amplitude and inter-event interval involved Mini Analysis software (Synaptosft, Decatur, GA, USA) and were compared by two-tailed unpaired Student’s *t*-test. The criterion for significance was *p* < 0.05.

## Results

### Increase of Self-Grooming Behavior in *Asic3*^−/−^ Mice

**Figure 1:**
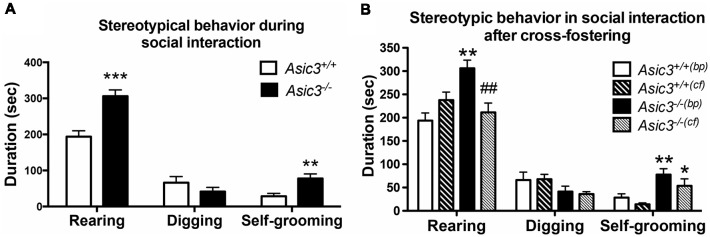
Increased self-grooming behavior during social interaction in *Asic3*^−/−^ mice. **(A)** *Asic3*^−/−^ mice displayed higher self-grooming and rearing behavior during juvenile social interaction compared to *Asic3*^+/+^ mice. Rearing: *t* = 4.699, *df* = 18, *p* < 0.0002; Self-grooming: *t* = 3.286, *df* = 18, *p* < 0.0041. *N* = 10 mice per group. **p < 0.01; ***p < 0.001. **(B)** Cross-fostering the *Asic3*^−/−^ mice to wild-type (WT) dam normalized the heightened rearing behavior in *Asic3*^−/−^ mice during social interaction, but did not change the level of grooming behavior. bp, biological parents; cf, cross-fostering. Rearing: genotype: *F*_(1,35)_ = 5.940, *p* = 0.020; Cross-fostering: *F*_(1,35)_ = 2.065, *p* = 0.160; interaction: *F*_(1,35)_ = 15.444, *p* < 0.001; *Post hoc* analysis: **p < 0.01 vs. *Asic3*^+/+(bp)^, ^##^*p* < 0.01 vs. *Asic3*^−/−(bp)^; Self-grooming behavior: genotype: *F*_(1,35)_ = 18.001, *p* < 0.001; Cross-fostering: *F*_(1,35)_ = 3.371, *p* = 0.075; interaction: *F*_(1,35)_ = 0.212, *p* = 0.648; *Post hoc* analysis: **p < 0.01 vs. *Asic3*^+/+(bp)^, *p < 0.05 vs. *Asic3*^+/+(cf)^. *N* = 9–10 mice per group. Data represent mean ± SEM. Data analyzed by two-tailed unpaired *t-test* **(A)** two-way analysis of variance (ANOVA; **B**).

Our prior work indicated that *Asic3*^−/−^ mice displayed increased stereotypic behavior, namely rearing and grooming behavior, in resident-intruder test (Wu et al., 2010). To understand whether the increase of stereotypic behavior is a general phenotype for *Asic3*^−/−^ mice or a phenotype induced by social stress, we examined the stereotypic behavior during reciprocal social interaction in juvenile *Asic3*^−/−^ mice with a same sex, strain, and similar age stranger mouse which is less stressful to the subject. Coincidentally, *Asic3*^−/−^ mice showed more rearing and self-grooming behavior than did *Asic3*^+/+^ mice (Figure 1A). This observation is consistent with our previous result from resident-intruder test.

We have previously demonstrated that *Asic3*^−/−^ mice had maternal caring deficit and thus affected the social development of their pups (Wu et al., 2009). To determine whether the grooming behavior phenotype is due to maternal stress produced by the *Asic3*^−/−^ dam, we cross-fostered *Asic3*^−/−^ offspring to WT dams. Cross-fostering did not alter grooming behaviors in either *Asic3*^+/+^ or *Asic3*^−/−^ mice (Figure 1B). The increase of grooming behavior remained pronounced in *Asic3*^−/−^ mice when cross-fostered to WT dams. In addition, we did not observe that maternal deficit leads to the increase of grooming behavior in *Asic3*^+/+^ mice (Figure 1B). On the contrary, the increase of rearing behavior was restored in *Asic3*^−/−^ mice when cross-fostered to WT dams. There’s a trend of increase of rearing behavior in *Asic3*^+/+^ after cross-fostering (Figure 1B). These results indicate that the increase of grooming behavior in *Asic3*^−/−^ is caused by genetic deletion, instead of environmental (e.g., maternal) impact.

**Figure 2:**
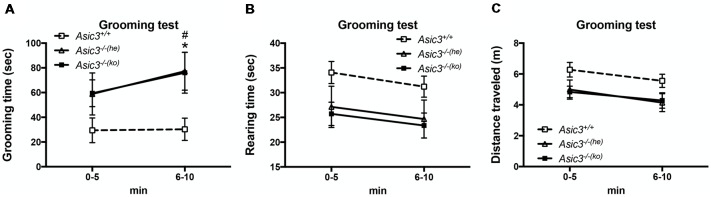
Increased self-grooming behavior in novel cage in *Asic3*^−/−^ mice. **(A)** The self-grooming behavior was increased in *Asic3*^−/−^ mice born to heterozygous dam (*Asic3*^−/−(he)^) and knockout dam (*Asic3*^−/−(ko)^). Data represent mean ± SEM. Genotype: *F*_(2,39)_ = 4.109, *p* = 0.0240; Time: *F*_(1,39)_ = 1.958, *p* = 0.1697; interaction: *F*_(2,39)_ = 0.4408, *p* = 0.6467; *Post hoc* analysis: *p < 0.05 *Asic3*^+/+^ vs. *Asic3*^−/−(he)^, ^#^*p* < 0.001 *Asic3*^+/+^ vs. *Asic3*^−/−(ko)^. **(B)** No change in the rearing behavior in the novel cage in *Asic3*^−/−^ mice. **(C)** No change in the distance traveled in the novel cage in *Asic3*^−/−^ mice. *N* = 13–15 mice per group. Data represent mean ± SEM. Data analyzed by two-way ANOVA.

To determine whether the increase of grooming behavior in *Asic3*^−/−^ mice can be triggered by novel environment instead of social stress, we performed grooming behavior test in a novel cage. Strikingly, *Asic3*^−/−^ mice exhibited more spontaneous grooming behavior than did *Asic3*^+/+^ mice (Figure 2A). Both *Asic3*^−/−^ born to *Asic3*^−/−^ mother (*Asic3*^−/−(ko)^) and *Asic3*^−/−^ born to *Asic3*^+/−^ mother (*Asic3*^−/−(he)^) displayed heightened grooming behavior in a novel cage. On the contrary, there was no difference in rearing behavior and locomotor activity between in *Asic3*^−/−(ko)^ and *Asic3*^−/−(he)^ mice in the novel cage (Figures 2B,C). We further conducted a digging behavior test, which is also considered a repetitive behavior, but *Asic3*^−/−^ mice did not show abnormal digging behavior (Supplementary Figure S1). Overall, these data strongly suggest that knockout of *Asic3* in mice causes the increase of grooming behavior. This phenotype is contributed by genetic deletion of *Asic3*, instead of maternal caring deficiency.

### Increased Dopamine Activity in Striatum in *Asic3*^−/−^ Mice

**Figure 3:**
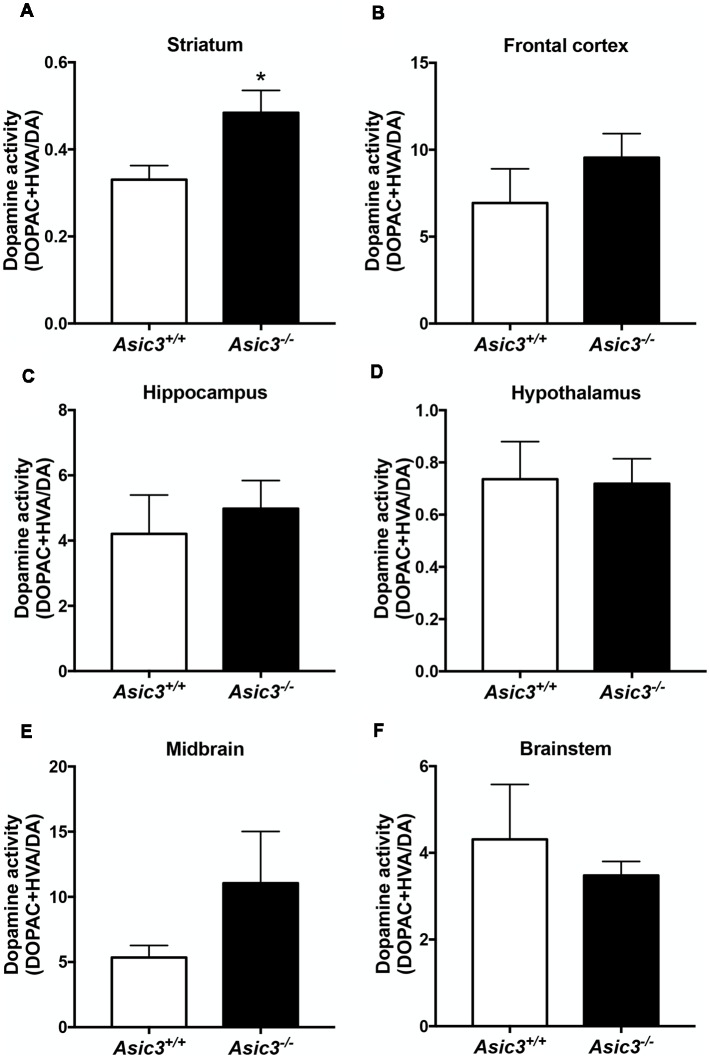
Measurement of dopamine (DA) activity in brain regions in *Asic3^−/−^* mice. **(A)** The DA activity [(DOPAC+HVA)/DA] was increased in striatum in *Asic3*^−/−^ mice as compared with *Asic3*^+/+^ mice: *t* = 2.532, *df* = 18, *p* < 0.0209; *p < 0.05. No difference was detected between *Asic3*^+/+^ and *Asic3*^−/−^ mice in **(B)** frontal cortex, **(C)** hippocampus, **(D)** hypothalamus, **(E)** midbrain, and **(F)** brainstem. *N* = 10 mice per group. Data represent mean ± SEM. Data analyzed by two-tailed unpaired *t-test*.

Prior studies implicate that brain DA is associated with grooming behavior in rodents (Kalueff et al., 2016). To understand whether the increase of grooming behavior in *Asic3*^−/−^ mice is associated with brain DA activity, we measured DA and its metabolites in different brain regions (Figure 3). Among the regions, only striatum showed difference in DA activity [(DOPAC+HVA)/DA] between *Asic3*^−/−^ and *Asic3*^+/+^ mice (Figure 3B). We did not detect any difference in DA activity between *Asic3*^−/−^ and *Asic3*^+/+^ mice in frontal cortex, hippocampus, hypothalamus, midbrain, and brainstem (Figure 3). This result indicates an altered DA activity in striatum in *Asic3*^−/−^ mice, which could be associated with the increase of grooming behavior.

### Elevation of Phosphorylated Extracellular Signal-Regulated Kinase 1/2 (ERK1/2) Activity in Dorsomedial Striatum and Bed Nucleus of the Stria Terminalis of *Asic3*^−/−^ Mice

**Figure 4:**
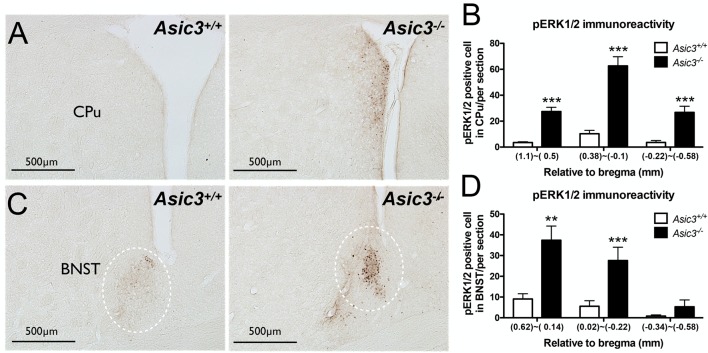
Phosphorylated extracellular signal-regulated kinase 1/2 (pERK) activity in dorsomedial striatum and bed nucleus of the stria terminalis (BNST) of *Asic3*^−/−^ and *Asic3*^+/+^ mice. **(A)** Representative images of pERK activation in striatal caudate putamen (CPu) in *Asic3*^+/+^ and *Asic3*^−/−^ mice. The pERK activity measured by pERK staining of brain sections revealed enhanced activation in striatum in *Asic3*^−/−^ mice. **(B)** Quantification of pERK^+^ cells in CPu. The pERK^+^ cells were significantly increased in CPu in *Asic3*^−/−^ mice as compared with *Asic3*^+/+^. *N* = 3–5 mice per group. (1.1~0.5mm, *Asic3*^+/+^ *n* = 36 slices, *Asic3*^−/−^ *n* = 36 slices, *t* = 7.191 *df* = 70; 0.38~-0.1mm, *Asic3*^+/+^ *N* = 24 slices, *Asic3*^−/−^ *N* = 18 slices, *t* = 7.661 *df* = 40; −0.22~-0.58mm, *Asic3*^+/+^ *n* = 16 slices, *Asic3*^−/−^ *n* = 14 slices, *t* = 5.012 df = 28, ***p < 0.001).** (C)** Representative images of pERK activation in BNST in *Asic3*^+/+^ and *Asic3*^−/−^ mice. **(D)** Quantification of pERK^+^ cells in BNST. The pERK^+^ cells were significantly increased in BNST in *Asic3*^−/−^ mice as compared with *Asic3*^+/+^ mice. *N* = 3–5 mice per group (0.62~0.13mm, *Asic3*^+/+^ *n* = 22 slices, *Asic3*^−/−^ *n* = 22 slices, *t* = 3.900 *df* = 42; 0.02~-0.22mm, *Asic3*^+/+^ *n* = 14 slices, *Asic3*^−/−^ *n* = 10 slices, *t* = 3.538 *df* = 22; -0.34~-0.58mm, *Asic3*^+/+^ *n* = 12 slices, *Asic3*^−/−^ *n* = 10 slices, **p < 0.01, ***p < 0.001). Data represent mean ± SEM. Data analyzed by two-tailed unpaired *t-test*.

pERK1/2 is a surrogate marker to examine neuronal activation in the nervous system (Gao and Ji, 2009). To understand whether the neuronal activity is abnormal in *Asic3*^−/−^ mice, we compared pERK immunoreactivity in the brain between genotypes. pERK1/2 immunoreactivity was increased in caudate putamen (CPu) and bed nucleus of the stria terminalis (BNST) in *Asic3*^−/−^ mice as compared with *Asic3*^+/+^ mice (Figure 4). The pERK1/2 immunoreactivity in CPu was mainly expressed in the dorsomedial part and was most abundant at 0.38–0.1 mm relative to bregma in *Asic3*^−/−^ mice, where pERK1/2 immunoreactivity was nearly absent in *Asic3*^+/+^ mice (Figures 4A,B). The pERK1/2 immunoreactivity in BNST was mainly expressed in the lateral part and was most abundant at 0.62–0.14 mm relative to bregma in *Asic3*^−/−^ mice, where pERK1/2 immunoreactivity was lower in *Asic3*^+/+^ mice (Figures 4C,D). Anatomically, dorsal striatum is one of the main regions controlling rodent self-grooming behavior (Kalueff et al., 2016). Lateral BNST is part of the extended amygdala, which is implicated in self-grooming behavior as well. Together, knockout of *Asic3* might alter the neuronal activity in the regions controlling self-grooming behavior.

### Perturbation of Synaptic Plasticity in Corticostriatal Circuits of *Asic3*^−/−^ Mice

**Figure 5:**
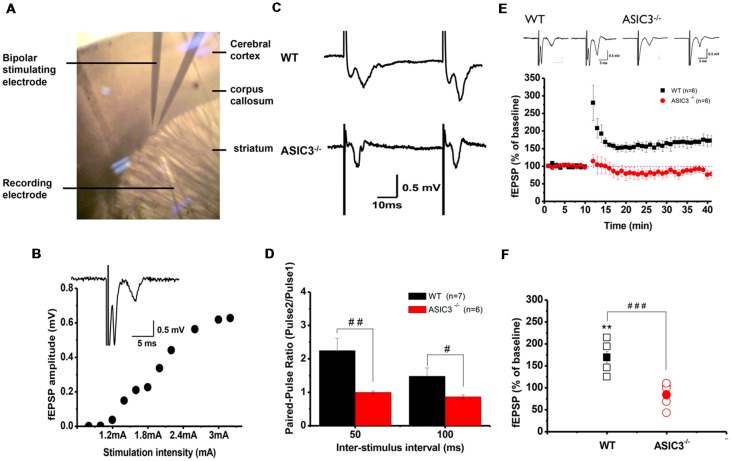
Electrophysiology properties of corticostriatal circuit in *Asic3*^−/−^ and *Asic3*^+/+^ mice. **(A)** Recording setup of corticostriatal field excitatory post-synaptic potentials (fEPSCs) in sagittal brain slices. The recording electrode was placed in the dorsal striatum, and a bipolar stimulating electrode was placed in the corpus callosum to evoke fEPSPs. **(B)** fEPSP evoked in cortico-striatal synapses, and fEPSP amplitude plotted as a function of stimulus intensity showing gradually growing responses with increasing intensity. Inset shows a typical field recording. **(C)** Tracings represent the paired pulse response at 50-ms interval. **(D)** The averaged paired-pulse ratios (PPRs) of *Asic3*^+/+^ and *Asic3*^−/−^ mice in 50- and 100-ms intervals (*N* = 6–7 mice per group). Paired pulse facilitation was significantly lower in *Asic3*^−/−^ mice than *Asic3*^+/+^ mice (50 ms: *t* = 2.99935, *df* = 11, *p* = 0.00605; 100 ms: *t* = 2.04147, *df* = 11, *p* = 0.03297; ^#^*p* < 0.05; ^##^*p* < 0.01). **(E)** High-frequency stimulation (HFS) of neocortical afferents could induce long-term potentiation (LTP) in the brain slices of *Asic3*^+/+^ animals. The cortico-striatal LTP was impaired in *Asic3*^−/−^ slices. Upper panel shows the representative tracings before (in left) and after (in right) HFS of *Asic3*^+/+^ and *Asic3*^−/−^ mice. **(F)** At 30 min after HFS, the mean fEPSP value was 84 ± 10% of pre-HFS values in *Asic3*^−/−^ mice and 169 ± 13% of pre-HFS values in *Asic3*^+/+^ mice (*Asic3*^+/+^: pre-HFS vs. HFS: *t* = 5.30428, *df* = 5, *p* = 0.00318; *Asic3*^+/+^ vs. *Asic3*^−/−^: *t* = 5.08298, *df* = 10, *p* = 0.00605, *p* < 0.001; *N* = 6–7 mice per group. **p < 0.01 vs. pre-HFS, ^###^*p* < 0.001 vs. *Asic3*^+/+^). Data are mean ± SEM. Data analyzed by two-tailed unpaired *t*-test.

Abnormalities in the frontal–striatal–thalamic circuitry are closely associated with stereotypic behaviors in rodent ASD or OCD models (Langen et al., 2011; Ting and Feng, 2011; Shepherd, 2013). To elucidate the functional consequences of disruption in ASIC3 activity on synaptic function, we focused on the corticostriatal pathway, which represents the most of the glutamatergic synapses in the striatum. We performed extracellular recordings of acute striatal brain slices from 8- to 10-week-old mice. A field recording electrode was placed in the dorso-lateral striatum and a bipolar stimulating electrode was placed nearby in the corpus callosum (Figure 5A) to evoke fEPSPs. Amplitude gradually increased with increasing stimulation from corticostriatal synapses in *Asic3^+/+^* brains (Figure 5B). We first examined the effect of *Asic3*^−/−^ on the PPR, a form of short-term plasticity that reflects the presynaptic function. The PPR for both interstimulus intervals 50 and 100 ms was significantly lower in *Asic3*^−/−^ than *Asic3*^+/+^ brains (Figures 5C,D). We next examined the effect of *Asic3*^−/−^ on long-term synaptic plasticity. After applying 3 trains of high frequency stimulation (HFS) at 100 Hz to *Asic3*^+/+^ brain slices, the fEPSP was significantly potentiated to 169 ± 13% of baseline, and the potentiation lasted for at least 30 min (Figure 5E). In contrast, the LTP was not induced in slices from *Asic3*^−/−^ mice (fEPSP = 84 ± 10% of baseline; Figure 5E). Therefore, loss of ASIC3 significantly impaired the corticostriatal LTP (Figure 5F), and the lack of LTP response is associated with a presynaptic impairment in synaptic transmission.

### Reduced Corticostriatal mEPSCs in *Asic3*^−/−^ MSNs

**Figure 6:**
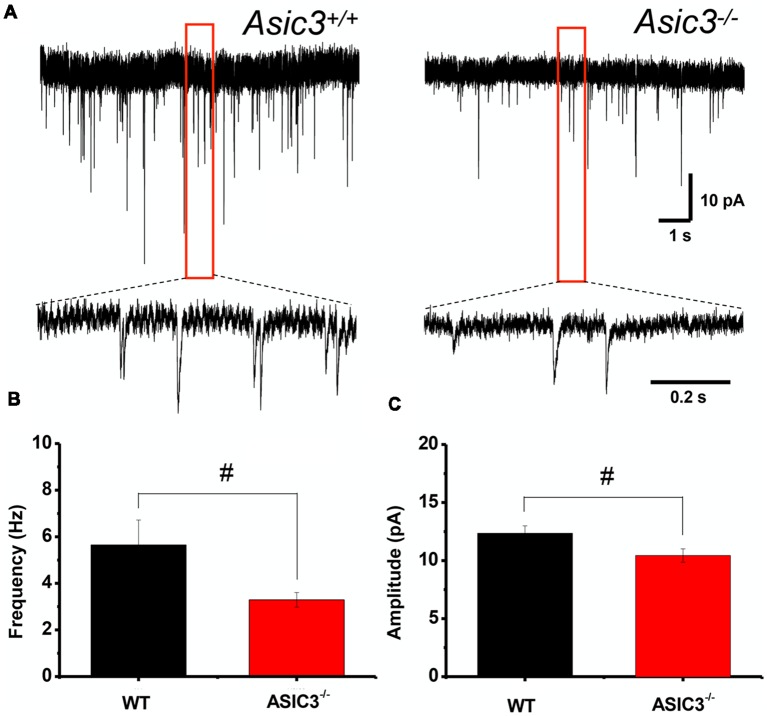
Reduced corticostriatal synaptic transmission in *Asic3*^−/−^ medium spiny neurons (MSNs). **(A)** Representative traces of mEPSC from *Asic3*^+/+^ and *Asic3*^−/−^ MSNs recorded with whole-cell voltage clamp. Reduced mEPSC: **(B)** the frequency and **(C)** the amplitude of mEPSC were reduced in *Asic3*^−/−^ MSNs as compared with *Asic3*^+/+^ MSNs (frequency: *t* = 2.27013, *df* = 16, *p* = 0.01869; amplitude *t* = 2.16085, *df* = 16, *p* = 0.02311; *N* = 8–10 mice per group; ^#^*p* < 0.05 vs. *Asic3*^+/+^). Data are mean ± SEM. Data analyzed by two-tailed unpaired *t*-test.

To further determine whether knockout of *Asic3* affects synaptic transmission in presynaptic or postsynaptic function, we performed whole-cell voltage clamp recording of mEPSCs in dorsolateral striatal MSNs. The frequency of mEPSCs was significantly lower in *Asic3*^−/−^ than *Asic3*^+/+^ MSNs (WT = 5.64 ± 1.07 Hz, *n* = 8 slices; *Asic3*^−/−^ = 3.29 ± 0.31 Hz, *n* = 10 slices; Figures 6A,B), which indicates defective presynaptic function in *Asic3*^−/−^ MSNs. As well, peak mEPSC amplitude was reduced in *Asic3*^−/−^ MSNs (WT = 12.35 ± 0.64 pA; *Asic3*^−/−^ = 10.43 ± 0.58 pA; Figures 6A,C), which indicates reduced postsynaptic response from the available synapses. Together, these data demonstrate a critical role for ASIC3 in modulating both presynaptic and postsynaptic function in corticostriatal circuitry.

### Low Dendritic Spine Density in *Asic3*^−/−^ MSNs

**Figure 7:**
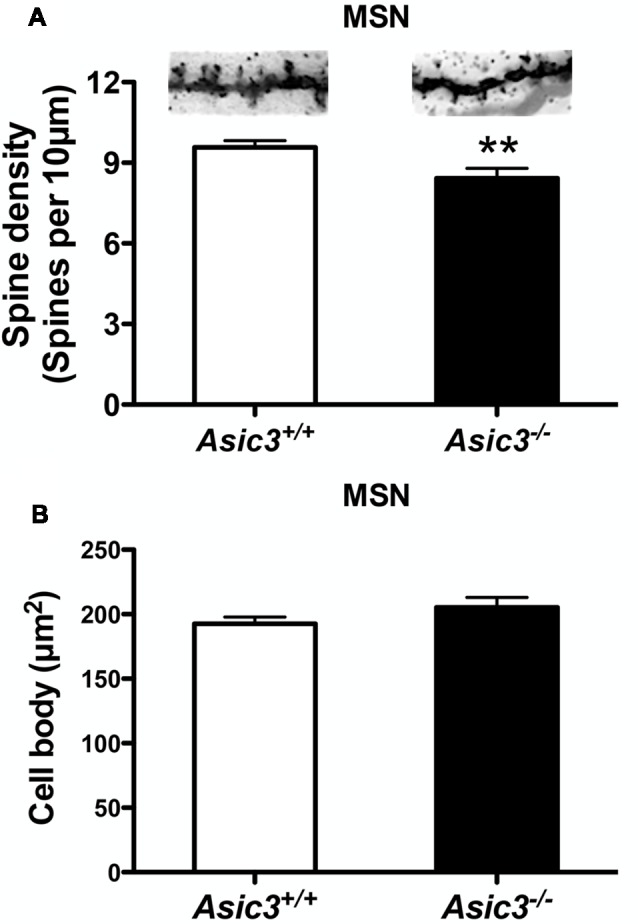
The effect of *Asic3* knockout on dendritic spines of striatal medium spiny neurons (MSNs). **(A)** The dendritic spine density of MSNs was lower in *Asic3*^−/−^ mice than in *Asic3*^+/+^ mice (*Asic3*^+/+^ *n* = 108 spines from six mice, *Asic3*^−/−^ *n* = 83 spines from five mice, *t* = 2.670 *df* = 189, *p* = 0.0082 by two-tailed *t*-test). **p < 0.01 vs. *Asic3*^+/+^. **(B)** No difference in MSNs cell body size between two genotypes (*Asic3*^+/+^ *n* = 118 spines from six mice, *Asic3*^−/−^ *n* = 92 from five mice). Data are mean ± SEM. Data analyzed by two-tailed unpaired *t*-test.

Reduction of dendritic spine is another feature indicating synaptic abnormality (Peça et al., 2011; Wang et al., 2016; Nagarajan et al., 2017). To investigate whether knockout of *Asic3* produces morphological changes in synapses in striatum, we performed Golgi staining to reveal the detailed synaptic structure in striatal MSNs. The density of striatal dendritic spines was significantly lower in *Asic3*^−/−^ mice as compared with *Asic3*^+/+^ mice (Figure 7A), with no difference in diameter of MSN cell bodies (Figure 7B). Consistent with the reduced corticostriatal synaptic transmission, we found low dendritic spine density in *Asic3*^−/−^ MSNs. The decreased dendritic spine density in *Asic3*^−/−^ MSNs might be associated with the reduced mEPSC in MSNs.

### Gene Expression of *Asic* Family in *Asic3*^−/−^ Mice

**Figure 8:**
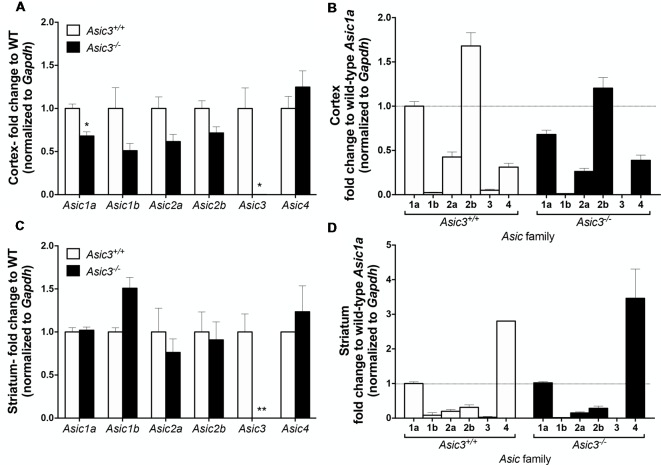
The gene expression of *Asic* subtypes in the cortex and striatum of *Asic3*^+/+^ and *Asic3*^−/−^ mice. The gene expression of *Asic1a*, *1b, 2a, 2b, 3* and *4* were analyzed by quantitative real-time polymerase chainreaction (qRT-PCR) in each brain region in each genotype. **(A)** Compared to the *Asic3*^+/+^ control for each gene, Lower expression of *Asic1a* and *Asic3* were detected in the cortex of *Asic3*^−/−^ mice. No difference was detected in other *Asic* subtypes between *Asic3*^+/+^ and *Asic3*^−/−^ mice in the cortex. *N* = 3 mice per group. *p < 0.05 vs. *Asic3*^+/+^. **(B)** Compared to the WT *Asic1a* control for each genotype. The distribution of *Asic* subtypes in the cortex of *Asic3*^+/+^ and *Asic3*^−/−^ mice. *N* = 3 mice per group. **(C)** Compared to the *Asic3*^+/+^ control for each gene, Lower expression of *Asic3* were detected in the striatum of *Asic3*^−/−^ mice. No difference was detected in other *Asic* subtypes between *Asic3*^+/+^ and *Asic3*^−/−^ mice in the striatum. *N* = 3 mice per group. *p < 0.05, **p < 0.01 vs. *Asic3*^+/+^. **(D)** Compared to the WT *Asic1a* control for each genotype. The distribution of *Asic* subtypes in the striatum of *Asic3*^+/+^ and *Asic3*^−/−^ mice. *N* = 3 mice per group. Data are mean ± SEM. Data analyzed by two-tailed unpaired *t*-test.

To understand whether deletion of *Asic3* alters the gene expression of other *Asic* subtypes, we analyzed *Asic1a, 1b, 2a, 2b, 3* and *4* expression in motor cortex and striatum by qRT-PCR. In cortex, deletion of *Asic3* slightly decreased *Asic1a* gene expression but did not affect other *Asic* subtypes expression (Figure 8A). Among these *Asic* subtype transcripts, cortex showed high levels of *Asic1a* and *Asic2b*, intermediate levels of *Asic2a* and *Asic4*, and nearly absence of *Asic1b* and *Asic3* in *Asic3*^+/+^ mice (Figure 8B). On the other hand, deletion of *Asic3* did not affect *Asic* subtypes expression in striatum (Figure 8C). Different from cortex, striatum showed high levels of *Asic1a* and *Asic4*, but low levels of *Asic1b*, *Asic2a*, *Asic2b*, and nearly absence of *Asic3* in *Asic3*^+/+^ mice (Figure 8D). Constitutional knockout of *Asic3* does not shift the expression pattern of *Asic* subtypes distribution in cortex and striatum (Figures 8B,D). The effect of *Asic3* knockout on other *Asic* family is minimal.

### Deletion of *Asic3* in Parvalbumin-Positive Neurons Increases Grooming Behavior

**Figure 9:**
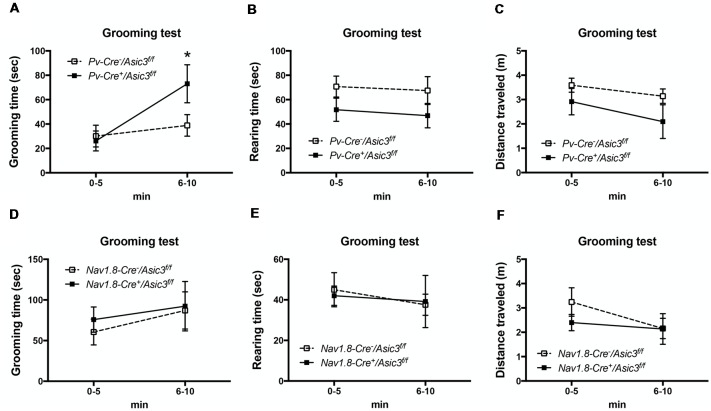
Deletion of *Asic3* in parvalbumin-positive (Pv^+^) neurons increased grooming behavior. **(A)** *Pv-Cre^+^/Asic3^f/f^* mice showed more grooming behavior than *Pv-Cre^−^/Asic3^f/f^* mice (genotype: *F*_(1,14)_ = 1.738, *p* = 0.2085; time: *F*_(1,14)_ = 7.431, *p* = 0.0164; interaction: *F*_(1,14)_ = 3.517, *p* = 0.0817; *Post hoc* analysis: *p < 0.05 0–5 min vs. 6–10 min in *Pv-Cre^+^/Asic3^f/f^*). *N* = 8 mice per group. No difference was found in **(B)** rearing and **(C)** distance traveled behaviors between in *Pv-Cre^+^/Asic3^f/f^* and *Pv-Cre^−^/Asic3^f/f^* mice. No difference was found in **(D)** grooming behavior, **(E)** rearing, and **(F)** distance traveled behaviors between *Na_v_1.8-Cre^+^*/*Asic3*^f/f^ and *Na_v_1.8-Cre^−^*/*Asic3*^f/f^ mice. *N* = 7–9 mice per group. Data are mean ± SEM. Data analyzed by two-way ANOVA.

Previously, our study demonstrated a role for ASIC3 in sensory mechanotransduction of proprioceptors (Lin et al., 2016). To test whether the increased grooming behavior of *Asic3*^−/−^ mice is correlated with the deficits in proprioception, we selectively knocked out ASIC3 expression in proprioceptors or nociceptors. We thus generated *Asic3* conditional knockout mice, with *Asic3* allele flanked by 2 loxP sites (*Asic3*^f/f^; Lin et al., 2016). *Asic3*^f/f^ mice were then crossed with *Pv-Cre* mice (Cre recombinase expressed in PV-positive neurons) to generate conditional *Asic3* knockout mice in parvalbumin-positive (PV^+^) neurons (*Pv-Cre^+^/Asic3^f/f^*). In somatosensory system, PV is a molecular marker specifically expressed in proprioceptors. We then examined the grooming behaviors in *Pv-Cre^+^/Asic3^f/f^* mice. Interestingly, *Pv-Cre^+^/Asic3^f/f^* mice showed increased grooming behavior as compared with *Asic3*^f/f^ without Cre recombinase (Figure 9A). In addition, *Pv-Cre^+^/Asic3^f/f^* mice showed lower rearing behavior and distance traveled (Figures 9B,C). Besides proprioceptors, ASIC3 is largely expressed in nociceptors, especially the neurons expressing Na_v_1.8, and involved in acid-induced chronic muscle pain (Chen et al., 2014). We next crossed *Asic3*^f/f^ and *Na_v_1.8-Cre* mice to selectively knockout ASIC3 in nociceptors (Nassar et al., 2004). Mice lacking ASIC3 in Na_v_1.8-positive neurons (*Na_v_1.8-Cre^+^*/*Asic3*^f/f^) showed normal grooming behavior (Figure 9D). Also, *Na_v_1.8-Cre^+^*/*Asic3*^f/f^ mice did not show other behavioral phenotypes as shown in *Pv-Cre*^+^/*Asic3^f/f^* mice (Figures 9E,F). Taken together, deletion of *Asic3* in PV-positive neuron could recapture the increase of grooming behavior phenotype we observed in *Asic3*^−/−^ mice, which support our hypothesis that the excess grooming behavior is caused by the deficits in proprioception.

## Discussion

In this study, we found that ASIC3 plays a significant role in mice grooming behavior. With constitutional knockout mice study, we observed the excessive grooming phenotype as well as enhanced striatum DA activity and pERK activation. Furthermore, *Asic3*^−/−^ mice showed neural plasticity changes in the corticostriatal circuit: lack of paired-pulse facilitation and LTP response, reduced mEPSCs in MSNs, and low dendritic spine density of MSNs. By using conditional knockout approaches, we identified ASIC3 expression in PV^+^-cells involved in the excessive grooming behavior of *Asic3*^−/−^ mice (Figure 9A).

ASDs are neurodevelopmental disorders characterized by impairments in social communications and interaction, as well as restricted and repetitive behaviors (Baron-Cohen and Belmonte, 2005). Although great majority of ASD research has focused on brain-specific mechanisms and circuits, accumulating evidence has shown abnormal sensory processing in ASD children (Robertson and Baron-Cohen, 2017). Strikingly, 60%–96% of ASD patients exhibit aberrant reactivity to sensory stimuli, especially with abnormal sensitivity to touch, proprioceptive, and painful stimuli (Suarez, 2012; Riquelme et al., 2016). Similar to the clinical observation of ASD, recent studies have shown aberrant neurosensory mechanotransduction in mouse models of ASD, including mutations in *Mecp2*, *Shank3b*, and *Fmr1* genes (Orefice et al., 2016). To understand the potential contribution of the PNS to ASD, David Ginty’s group has conducted a comprehensive genetic study in mouse models of ASD and discovered the expression of *Mecp2* in peripheral somatosensory neurons can restore the ASD-like deficit in *Mecp2*^−/y^ mice. Although their study suggests a role for the neurosensory mechanotransduction in the development of ASD, the specific subsets of mechanosensory neurons and the molecular transducers that contribute to ASD phenotypes are still largely unknown.

Here, we provided evidence that ASIC3 of proprioceptors could be one of the candidates that contributes to the grooming phenotypes related to ASD. Especially, the conditional knockout of ASIC3 in PV^+^ cells recaptured the excessive grooming phenotypes of whole-body *Asic3* knockout. ASIC3 is one of the most sensitive acid-sensitive ion channels predominantly expressed in somatosensory neurons that are responsible for pain and proprioception (Cheng et al., 2018). PV is a calcium-binding protein specifically expressed in interneurons of the CNS and in proprioceptors of the PNS. Our prior study showed there is no expression of ASIC3 in the PV^+^ proprioception related nuclei in the brain (e.g., cerebellum or precerebellar nuclei) by using ASIC3 GFP reporter mice (Lin et al., 2016). On the contrary, ASIC3 predominately express in trigeminal ganglion (TG) and dorsal root ganglion (DRG; Ichikawa and Sugimoto, 2002; Lin et al., 2016). We further identified that ASIC3 colocalized with PV^+^ neurons in DRG (Lin et al., 2016). Evidence to show the expression of ASIC3 in the CNS is minimal. Most studies were either based on PCR or immunohistochemistry and were failed to show negative control in knockout mice (Lin et al., 2016). Therefore, proprioceptors could be the PV^+^-cells in the peripheral that contribute to the grooming phenotypes in *Asic3*^−/−^ mice. Synaptic tracing techniques could be one of the methods to understand the peripheral inputs from DRG or TG to corticostriatal circuits.

One would be curious about where and how loss of ASIC3 activation in proprioceptors could contribute to the modulation of grooming behaviors. The developmental compensation due to abnormal proprioceptive inputs could account for the circuitry changes in *Asic3*^−/−^ brain, although we cannot exclude the contribution of ASIC3 in the CNS. *Asic3*^−/−^ mice showed increased DA turnover rate and pERK activation in their striatum, which could be related with each other. DA activates pERK through the D1 receptor in MSNs. Stimulation of the D1 receptor activates cAMP/PKA and further phosphorylates DARPP-32, which inhibits dephosphorylation of pERK1/2 to ERK by regulating striatal enriched phosphatase (STEP; Shiflett and Balleine, 2011). Moreover, upregulation of Ras/Raf/ERK1/2 signaling was found in the frontal cortex of human ASD patients and an ASD mouse model, BTBR (Yang et al., 2011; Zou et al., 2011). Mounting evidence also suggests an association of dysfunctional ERK/PI3K signaling and ASD (Levitt and Campbell, 2009). Further research is needed to elucidate the mechanism of ASIC3 affecting DA signaling and ERK activity. Other neural transmission associated signaling cascades (e.g., pCREB, PKA) or immediate early genes (e.g., c-Fos, EGR1, ARC) respond to specific external stimuli could also serve as probes to further identify the grooming-associated molecular changes.

The electrophysiology properties and low dendritic spine density in striatum of *Asic3*^−/−^ mice support the hypothesis that grooming behavior is closely related to cortico–striatal circuit in rodents (Herbert, 2011). The impaired paired-pulse facilitation and LTP response in corticostriatal circuits of *Asic3*^−/−^ mice highlights the weakened MSN synaptic plasticity. Although most previous studies showed that postsynaptic proteins in excitatory neurons contribute significantly to the grooming phenotype (Welch et al., 2007; Peça et al., 2011), reduced PPR and frequency of mEPSCs in *Asic3*^−/−^ mice suggest that the presynaptic component might also participate in regulating grooming behavior. Both presynaptic and postsynaptic neurons in the corticostriatal circuit may contribute in concert to excessive grooming.

Previous study showed that most ASIC-like currents in mouse striatal MSNs are contributed by homomeric ASIC1a and heteromeric ASIC1a+2 (Jiang et al., 2009). The pH_50_ in MSNs was about 6.25, close to that observed in ASIC1a homomeric channels. The acid-evoked currents were absent in >90% MSNs from *Asic1a*^−/−^ mice. Treatment with amiloride (common ASIC blocker) and PcTX1 (selected ASIC1a antagonist) largely reduced acid-evoked currents in most MSN neurons (70.5%). The remaining neural population expressed heteromeric ASIC1a+2 channels based on PcTx1 and Zn sensitivity (Jiang et al., 2009). The possibility that ASIC3 expression comprises heteromeric ASIC channels with ASIC1a is low, because we could not amplify full-length ASIC3 transcripts in striatum (Wu et al., 2010). Even the ASIC3 transcripts are detectable in the brain in some studies, there is no evidence showing ASIC3 is functional in the brain.

The reduced dendritic spine density in *Asic3*^−/−^ MSNs is unexpected, because most MSN ASIC channels contain ASIC1a. *Asic3*^−/−^ mice may have a compensatory mechanism that alters the postsynaptic ASIC1a expression levels in MSNs during neural development. In hippocampus organotypic slices, overexpressing ASIC1a increased dendritic spine number and* vice versa* (Zha et al., 2006). The same mechanism may affect ASIC1a level in postsynaptic striatal MSNs and further alter dendritic spine density in mice without ASIC3. Accordingly, the decreased dendritic spine density in *Asic3*^−/−^ MSNs is associated with low mEPSC amplitude observed in the same mice.

Does ASIC3 of sensory neurons directly respond to grooming stimuli? Grooming behavior requires the facial perception of mechanistic stimuli. ASIC3 belongs to the ENaC/Deg family and is structurally related to degenerin mechanoreceptors (Coscoy and Barbry, 2004; Kang et al., 2012). ASIC3 channels in mice are distributed at sensory nerve endings of skin, periodontal ruffini endings of incisors, and TG, all of which could act together at the oro-facial area to transmit the signals of mechanical stimuli into the CNS (Chen and Wong, 2013). The mechanosensation and proprioception of the oro-facial area could be altered in *Asic3*^−/−^ mice based on our previous findings (Lin et al., 2016). PV-producing neurons are fast-spiking GABAergic interneurons or primary sensory neurons of the mesenteric trigeminal nucleus (Me5) that innervate the jaw-closing muscle spindles (Lazarov, 2007). The acid-evoked properties in Me5 neurons differ from those in TG and DRG (Connor et al., 2005). The PV^+^ interneurons generate gamma oscillations that are impaired in patients with ASD or schizophrenia (Sohal et al., 2009; Lewis et al., 2012). Loss of *Asic3* may diminish the sense of proprioception and thus reduce awareness of the grooming stimuli.

Here, we hypothesize that the excessive grooming phenotype is contributed by the peripheral ASIC3 in PV^+^ cell population, likely DRG or TG, based on our previous work (Lin et al., 2016). Furthermore, the grooming behavioral abnormality is contributed by PV^+^ proprioceptors, instead of Nav1.8^+^ nociceptors. The functionality of ASIC3 in the CNS is still under debate. To date, we showed almost no expression of *Asic3* in the striatum in the WT mice (Figure 8D). Also, the gene compensation effects were minimal in the cortex and striatum of *Asic3*^−/−^ mice and thus unlikely contributed to the grooming phenotypes. Nevertheless, further studies are needed to further validate the causal effect of proprioceptor ASIC3 knockout and grooming phenotype. The neuronal and synaptic dysfunction we observed in *Asic3*^−/−^ mice were consistent with other genetic engineered mice with excessive grooming behavior, such as *Shank3* mutant mice (Peça et al., 2011; Wang et al., 2011; Fuccillo, 2016). The convergent behavioral, neuronal, and synaptic phenotypic observations implicate the grooming behavior is likely an outcome of striatal synaptic dysfunction. Other regions associated with corticostriatal circuit and grooming behavior (e.g., amygdala and thalamus) might also involve. The developmental alteration of morphological, neurochemical, and functional consequence in the corticostriatal circuitry in *Asic3* constitutional and conditional knockout mice is warranted.

In conclusion, we have demonstrated that ASIC3 plays an important role in mouse grooming behavior. Multidisciplinary approaches indicated that knockout of ASIC3 changed striatal characteristics. Conditional knockout techniques further demonstrate that ASIC3 excision in PV^+^ neurons led to the increase of self-grooming by mice. ASIC3 knockout may alter the corticostriatal circuit and thus affect grooming behavior in mice. Research into ASIC3 and psychiatric-related behavior with *Asic3*^−/−^ mice can be a valuable direction to assist translational research into neuropsychiatric disorders.

## Author Contributions

W-LW conducted and analyzed behavior experiments, immunohistochemistry, HPLC analysis, Golgi staining and gene expression analysis. S-JC performed and analyzed results from all electrophysiology experiments. S-HL generated and verified *Asic3* conditional knockout mice. Y-CC analyzed dendritic spines in Golgi stain and performed gene expression experiment. EY-KH assisted in DA activity measurement. W-LW and C-CC collected, integrated, and interpreted the results and wrote the manuscript.

## Conflict of Interest Statement

The authors declare that the research was conducted in the absence of any commercial or financial relationships that could be construed as a potential conflict of interest.

## Supplementary Material

FIGURE S1 Deletion of Asic3 showed no effect on digging behavior. The digging behavior was not changed in *Asic3^−/−^* mice born to heterozygous dam (*Asic3^−/−(he)^*) and knockout dam (*Asic3^−/−(ko)^*). Data are mean ± SEM. Data analyzed by one-way ANOVA.
