# Supplementary figures and images for: The Effect of ASIC3 Knockout on Corticostriatal Circuit and Mouse Self-grooming Behavior
Source: Front Cell Neurosci. 2019 Mar 12;13:86. doi: 10.3389/fncel.2019.00086 (PMC6424217; doi:10.3389/fncel.2019.00086)

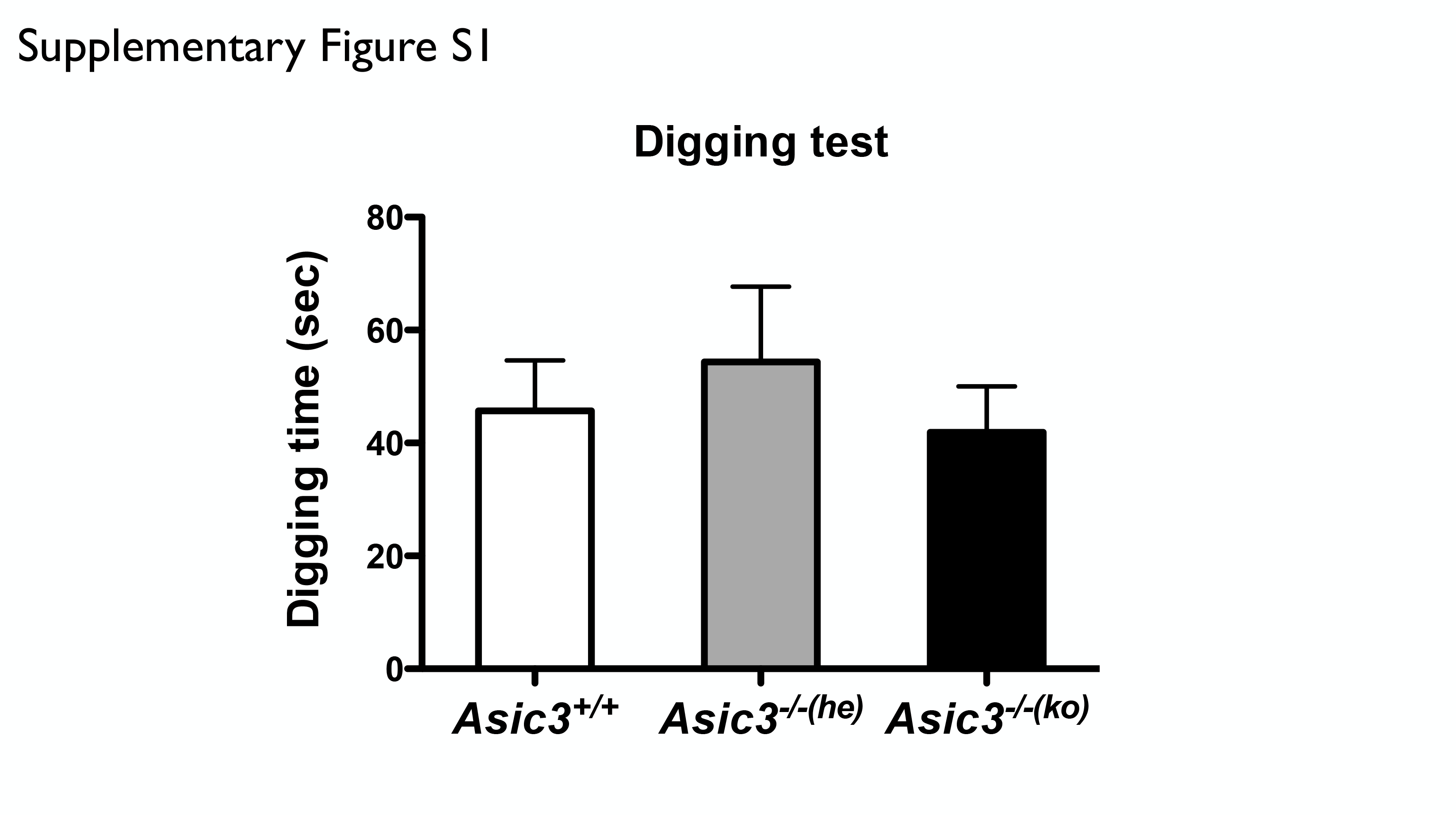

Supplement: FIGURE S1 — Deletion of Asic3 showed no effect on digging behavior. The digging behavior was not changed in Asic3−/− mice born to heterozygous dam (Asic3−/−(he)) and knockout dam (Asic3−/−(ko)). Data are mean ± SEM. Data analyzed by one-way ANOVA. [file Image_1.TIF]
